# Scoping Review of Antimalarial Drug Candidates in Phase I and II Drug Development

**DOI:** 10.1128/aac.01659-21

**Published:** 2022-02-15

**Authors:** Azrin N. Abd-Rahman, Sophie Zaloumis, James S. McCarthy, Julie A. Simpson, Robert J. Commons

**Affiliations:** a QIMR Berghofer Medical Research Institute, Brisbane, Queensland, Australia; b Biostatistics Unit, Centre for Epidemiology and Biostatistics, Melbourne School of Population and Global Health, University of Melbourne, Parkville, Victoria, Australia; c The Peter Doherty Institute for Infection and Immunity, The University of Melbourne and the Royal Melbourne Hospital, Melbourne, Victoria, Australia; d Global Health Division, Menzies School of Health Research and Charles Darwin University, Darwin, Northern Territory, Australia; e Internal Medical Services, Ballarat Health Services, Ballarat, Victoria, Australia

**Keywords:** antimalarial, phase 1, phase 2, drug development, malaria

## Abstract

The emergence and spread of parasite resistance to currently available antimalarials has highlighted the importance of developing novel antimalarials. This scoping review provides an overview of antimalarial drug candidates undergoing phase I and II studies between 1 January 2016 and 28 April 2021. PubMed, Web of Science, Embase, clinical trial registries, and reference lists were searched for relevant studies. Information regarding antimalarial compound details, clinical trial characteristics, study population, and drug pharmacokinetics and pharmacodynamics (PK-PD) were extracted. A total of 50 studies were included, of which 24 had published their results and 26 were unpublished. New antimalarial compounds were evaluated as monotherapy (28 studies, 14 drug candidates) and combination therapy (9 studies, 10 candidates). Fourteen active compounds were identified in the current antimalarial drug development pipeline together with 11 compounds that are inactive, 6 due to insufficient efficacy. PK-PD data were available from 24 studies published as open-access articles. Four unpublished studies have made their results publicly available on clinical trial registries. The terminal elimination half-life of new antimalarial compounds ranged from 14.7 to 483 h. The log_10_ parasite reduction ratio over 48 h and parasite clearance half-life for Plasmodium falciparum following a single-dose monotherapy were 1.55 to 4.1 and 3.4 to 9.4 h, respectively. The antimalarial drug development landscape has seen a number of novel compounds, with promising PK-PD properties, evaluated in phase I and II studies over the past 5 years. Timely public disclosure of PK-PD data is crucial for informative decision-making and drug development strategy.

## INTRODUCTION

Malaria is a debilitating mosquito-borne infectious disease caused by parasites of the *Plasmodium* family. Despite progress in malaria control, it remains a major public health problem, with 229 million clinical cases and 409,000 deaths globally in 2019 (1). Due to its high morbidity and mortality, malaria places a social and economic burden on many developing countries. Concerted efforts are needed to accelerate progress toward malaria elimination to achieve the target of reducing global malaria incidence and mortality rates by at least 90% by 2030 (2).

One of the threats for malaria elimination is the emergence of parasites resistant to currently available antimalarials. The emergence of chloroquine-resistant Plasmodium falciparum was first discovered in the Greater Mekong subregion and spread independently through Asia, South America, and Africa (3). First reported in 1989, evidence of chloroquine-resistant Plasmodium vivax has accumulated steadily in many countries of endemicity (4). More recently, P. falciparum resistance to artemisinin derivatives and partner drugs was identified in western Cambodia (5, 6) before spreading through Southeast Asia (7–9), leading to significant rates of treatment failure for these widely used artemisinin-based combination therapies.

The unrelenting rise of multidrug-resistant malaria demands the continuous development of novel antimalarial compounds. This process of development extends from preclinical studies to early clinical trials in human volunteers, and then to phase III clinical trials in patients, with the goal of achieving drug registration and availability in areas of endemicity. Transition through this pipeline can take many years. There are several antimalarial drug candidates currently being evaluated in phase II studies (10). However, the probability that drugs will progress to licensure depends on many factors, including pharmacokinetic (PK) profile, pharmacodynamic (PD) effect, safety, susceptibility to generation of resistance (11), and transmission-blocking properties.

The urgent need for novel antimalarial medicines has resulted in an increase in new chemical entities entering clinical development. Despite the increasing number of phase I and II studies, there has been a lack of reviews underlining the progress made by antimalarial drug candidates in the drug development pipeline. As phase I and II studies result in the collection of important safety and efficacy data, an understanding of drug PK and PD is essential for guiding the selection of antimalarial therapies to progress to pivotal phase III studies. Thus, we conducted a scoping review to summarize findings of antimalarial drug candidates undergoing evaluation in phase I and II studies.

The objectives of this scoping review were to (i) compile a collection of antimalarial drug candidates under investigation in phase I and II studies, and (ii) collate PK and PD data for identified antimalarial drug candidates.

### Eligibility criteria.

The current scoping review was conducted using the Joanna Briggs Institute *Manual for Evidence Synthesis Methodology* (61), and the results were reported according to the Preferred Reporting Items for Systematic Reviews and Meta-Analyses extension for Scoping Reviews (PRISMA-ScR) tool (62) (Table S1 in the supplemental material).

Studies were eligible if they were studies involving antimalarial compounds under investigation in phase I and II studies for treatment of any species of malaria, registered or published between the period of 1 January 2016 and 28 April 2021, written in all languages, and involved human participants. Studies were excluded if the studies involved vaccine candidates, antimalarial compounds used for malaria chemoprophylaxis or nonmalaria treatment, or information on the PK and parasitemia clearance as well as full-text were not available (for published studies).

A new antimalarial compound is defined as a drug (or a drug combination) that is not previously registered for use in human malaria. Although tafenoquine has been approved for treating the liver stage of vivax malaria and malaria prophylaxis, we have included studies of tafenoquine where its role in clearance of asexual blood-stage infection and transmission reduction in falciparum malaria has been evaluated.

### Information sources.

The following databases and clinical trial registries were searched initially on 11 January 2021 to identify potentially relevant studies: PubMed, Web of Science, Embase, clinicaltrials.gov, and the International Clinical Trials Registry Platform. Identification of additional compounds was performed through the global portfolio of antimalarial medicines on the Medicines for Malaria Venture (MMV) website (https://www.mmv.org/research-development/mmv-supported-projects, assessed initially on 24 March 2021), as well as from reference lists from identified studies and review articles. Searches were updated to include studies published or registered up to 28 April 2021.

### Search strategy.

A two-step literature ‘search was performed independently by two authors (A.N.A.-R. and R.J.C.). The search terms malaria OR plasmodi* OR antimalarial AND (“phase 1” OR “phase 2” OR “phase 2a” OR “phase 2b”) were used in the first-step search. In the second step, each identified antimalarial was then searched individually on the same databases and clinical trial registries by name(s), for example, ((KAF156 OR GNF156 OR ganaplacide) AND (malaria OR plasmodi*)).

### Selection of sources of evidence.

Two authors (A.N.A.-R. and R.J.C.) evaluated the titles and abstracts of the potentially relevant studies identified by the search. Full texts of published studies were then retrieved by one author (A.N.A.-R.) and reviewed for inclusion. Studies that did not satisfy the eligibility criteria were excluded and classified according to reason for exclusion.

### Data charting process.

One author (A.N.A.-R.) extracted the relevant information from eligible studies using a standardized form. The design of this extraction form was initially piloted by three authors (A.N.A.-R., R.J.C., and S.Z.) for six compounds and refined with input from all coauthors.

### Data items.

The data on antimalarial compound details (compound name, dose, route of administration), clinical trial characteristics (registration number, phase, status, country), study population, sample size, PK, and parasite clearance information were extracted for each study by a single author (A.N.A.-R.). Extracted values were recorded in standard units.

### Synthesis of results.

Data were explored using narrative analysis. Studies were organized and described by type of therapy (monotherapy or combination therapy), status of antimalarial compounds in drug development pipeline, and data availability. A narrative synthesis of PK-PD parameter estimates of new antimalarial compounds was also performed.

### Search results.

**FIG 1:**
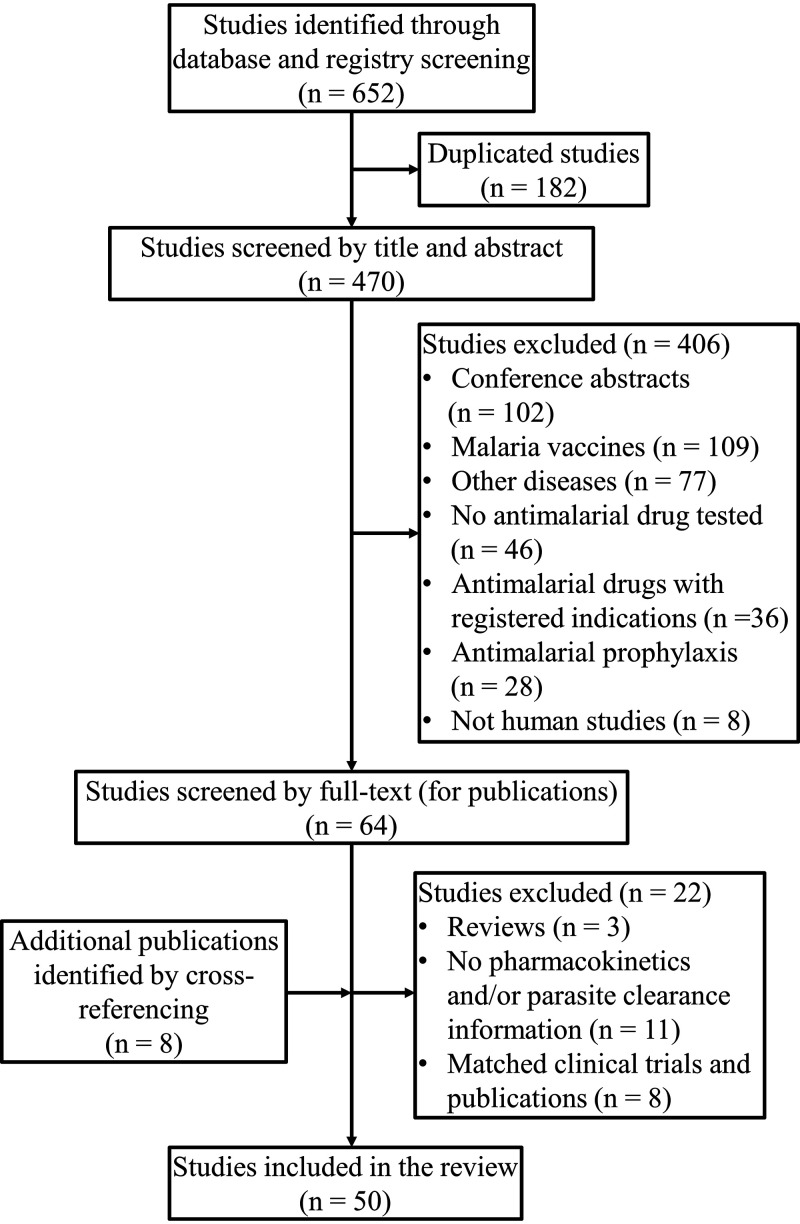
Search strategy flowchart.

Approximately 652 studies were returned from the database and clinical trial registry search (Fig. 1). After removing duplicates (*n* = 182), 470 studies were screened by titles and abstracts. Following the screening process, 406 studies were excluded for reasons detailed in Fig. 1. Studies that did not meet eligibility criteria were excluded after full-text review (*n* = 22). An additional eight studies were identified by cross-referencing. As a result, 50 studies were included in this review.

### Characteristics of included studies.

There were 27 phase I, 20 phase II, and 3 combined phase I and II studies for 31 antimalarial drug candidates. Of the included studies, 24 had published their results, while the remaining 26 were not published. For the unpublished studies, 12 had completed the recruitment, 6 were in the process of recruiting, 3 were terminated, 2 were withdrawn, 2 had not started recruitment, and the status of 1 study was unknown (Table 1). Key information of published studies is presented in Table 2. The phase I studies enrolled between 6 and 72 participants, and the phase II studies recruited 8 to 437 participants.

**TABLE 1:** Registered phase I and II clinical trials for drug candidates for treatment of malaria that have not published their findings (1 January 2016 to 28 April 2021)^*f*^

Compound	Clinical trial ID	Phase	Status	Study population	Site(s)	Organization(s)
Artefenomel (formerly OZ439)	NCT04069221	I	Completed	Healthy volunteers	Netherlands	MMV
Cipargamin (formerly KAE609, NIDT609)	NCT03334747	II	Completed	Uncomplicated P. falciparum malaria adult patients	Gabon, Ghana, Mali, Rwanda, Uganda	Novartis Pharmaceuticals, Wellcome Trust
	NCT04321252^*a*^	I	Completed	Healthy volunteers	Belgium	Novartis Pharmaceuticals, Wellcome Trust
DM1157	NCT03490162	II	Terminated (toxicity in higher dose groups and a therapeutic dose level was not found in lower dose groups)	Healthy volunteers	United States	National Institute of Allergy and Infectious Disease
DSM265	NCT03637517^*b*^	I	Completed	Healthy volunteers	United States	MMV, AbbVie
	NCT02750384^*c*^	I	Terminated (sponsor strategic decision based on preliminary results)	Healthy volunteers	United States	MMV, AbbVie
M5717 (formerly DDD107498; DDD498; MMV121)	NCT03261401	I	Completed	Healthy volunteers and P. falciparum-infected healthy subjects	Australia	Merck KGaA
Meplazumab	NCT04327310	I	Not yet recruiting	Healthy volunteers and P. falciparum-infected healthy subjects	Not provided	Jiangsu Pacific Meinuoke Bio Pharmaceutical Co., Ltd.
MMV390048 (also known as MMV048)	NCT02880241	II	Terminated (sponsor strategic decision)	Uncomplicated P. falciparum or P. vivax malaria adult patients	Ethiopia	MMV, University of Gondar, Jimma University
MMV688533 (also known as MMV533, SAR441121)	NCT04323306	I	Recruiting	Healthy volunteers	Australia	MMV, Nucleus Network Ltd., Southern Star Research Pty Ltd
SAR441121	ACTRN12618001783213	I	Recruiting (trial has not been updated in >2 yrs)	Healthy volunteers and P. falciparum-infected healthy subjects	Australia	Sanofi-Aventis R&D
(+)-SJ000557733 (also known as SJ733)	NCT04709692 and PER-045-20^*d*^	II	Recruiting	Uncomplicated P. falciparum or P. vivax malaria adult patients	Peru	R. Kiplin Guy, Global Health Innovative Technology Fund, Eisai Inc., Asociacion Civil Selva Amazonica
Tafenoquine	NCT04609098	II	Completed	Uncomplicated P. falciparum pediatric and adult patients	Mali	London School of Hygiene and Tropical Medicine
	ACTRN12620000995976	I	Recruiting	P. falciparum-infected healthy subjects	Australia	Bill and Melinda Gates Foundation
ZY-19489 (formerly AZ13721412; MMV674253)	ACTRN12619000127101	I	Completed	Healthy volunteers	Australia	Cadila Healthcare Limited
	ACTRN12619001466134^*e*^	I	Completed	Healthy volunteers	Australia	Cadila Healthcare Limited
	ACTRN12619001215112	I	Completed	P. falciparum-infected healthy subjects	Australia	Cadila Healthcare Limited
5-ALA HCl with SFC	NCT04020653	II	Withdrawn (Considering the FDA Thailand requirement, changes of malaria cases in Thailand, and ethic committee-recommendation)	Uncomplicated P. falciparum malaria adult patients	Thailand	Neopharma Japan Co., Ltd.
	CTRI/2018/09/015824	II	Not yet recruiting (trial has not been updated in >2 yrs)	Uncomplicated P. falciparum malaria adult patients	India	Neopharma Japan Co., Ltd
Artefenomel-ferroquine	NCT03660839	II	Completed	Uncomplicated P. falciparum pediatric and adult patients	Benin, Burkina Faso, Gabon, Kenya, Uganda	Sanofi, MMV
Artefenomel-piperaquine	NCT03542149	I	Completed	P. falciparum-infected healthy subjects	Australia	MMV, QIMR Berghofer Medical Research Institute, Clinical Network Services (CNS) Pty Ltd., Q-Pharm Pty Limited
Ganaplacide with LUM-SDF	NCT04546633	II	Recruiting	Uncomplicated P. falciparum malaria pediatric patients	Mali	Novartis Pharmaceuticals, European and Developing Countries Clinical Trials Partnership
	NCT03167242	II	Recruiting	Uncomplicated P. falciparum malaria pediatric and adult patients	Burkina Faso, Côte D'Ivoire, Gabon, Gambia, India, Kenya, Mali, Mozambique, Thailand, Uganda, Vietnam	Novartis Pharmaceuticals, MMV
Imatinib-DHA-piperaquine	NCT03697668	II	Unknown (trial has not been updated in >2 yrs)	Uncomplicated P. falciparum malaria adult patients	Vietnam	Nurex S.r.l., University of Sassari, Purdue University, Vinmec Healthcare System
Methylene blue with artemether and lumefantrine	NCT02696928	II	Withdrawn (lack of ethical approval in Ethiopia)	P. vivax malaria adult patients	Ethiopia	Heidelberg University, Ludwig Maximilians University of Munich, Jimma University
Ruxolitinib-artemether-lumefantrine	NCT04456634	I	Completed	Healthy volunteers	Australia	MMV, Southern Star Research Pty Ltd., Nucleus Network Ltd

a Administered intravenously.

b DSM265-TGPS (tocopheryl polyethylene glycol succinate) 34% SDD (spray dried dispersion) granules formulation in comparison with a reference DSM265 25% SDD powder for suspension formulation.

c DSM265 50% SDD granules formulation in comparison with a reference DSM265 25% SDD powder for suspension formulation.

d Administered in combination with or without cobicistat.

e Administered with a high-fat meal.

f Clinical trials registered between 1 January 2016 and 28 April 2021 that have published their findings are listed in Table 2. 5-ALA HCl, 5-aminolevulinic acid hydrochloride; SFC, sodium ferrous citrate; FDA, Food and Drug Administration; LUM-SDF, lumefantrine solid dispersion formulation; DHA, dihydroartemisinin; MMV, Medicines for Malaria Venture.

**TABLE 2:** Published phase I and II clinical trials for drug candidates for treatment of malaria (1 January 2016 to 28 April 2021)^*a*^

Compound	Clinical trial ID	Phase	Study population	No. of subjects	Site(s)	Organizations	Ref
ACT-451840	ACTRN12614000781640	I	P. falciparum-infected healthy subjects	8	Australia	Actelion Pharmaceuticals Australia Pty.	(12)
AQ-13	NCT01614964	II	Uncomplicated P. falciparum malaria adult patients	33	Mali	Tulane School of Public Health and Tropical Medicine, University of the Sciences, Techniques and Technologies of Bamako	(13)
Artefenomel (formerly OZ439)	NCT02573857	I and II	P. vivax-infected healthy subjects	8	Australia	MMV, Clinical Network Services (CNS) Pty Ltd., Q-Pharm Pty Limited, QIMR Berghofer Institute of Medical Research	(14)
	ACTRN12612000814875	II	P. falciparum-infected healthy subjects	24	Australia	MMV, QIMR Berghofer Institute of Medical Research	(15)
	NCT01213966	II	Uncomplicated P. falciparum and P. vivax malaria adult patients	82	Thailand	MMV, Mahidol University	(16)
Cipargamin (formerly KAE609, NIDT609)	Not provided	I	Healthy volunteers	6	Netherland	Novartis Pharmaceuticals	(17)
	NCT02543086	I	P. falciparum-infected healthy subjects	8	Australia	Novartis Pharmaceuticals, MMV	(18)
	NCT01836458	II	Uncomplicated P. falciparum malaria adult patients	25	Vietnam	Novartis Pharmaceuticals	(19)
DSM265	ACTRN12613000522718 and ACTRN12613000527763	I	Healthy volunteers and P. falciparum-infected healthy subjects	62	Australia	MMV, QIMR Berghofer Institute of Medical Research, CPR Pharma Services	(20)
	NCT02573857	I	P. falciparum-infected healthy subjects	8	Australia	MMV, Clinical Network Services (CNS) Pty Ltd., Q-Pharm Pty Limited, QIMR Berghofer Institute of Medical Research	(21)
	NCT02123290	II	Uncomplicated P. falciparum and P. vivax malaria adult patients	45	Peru	MMV, Asociacion Civil Selva Amazonica	(22)
Ferroquine (formerly SSR97193, ferrochloroquine)	ACTRN12613001040752	II	P. falciparum-infected healthy subjects	8	Australia	MMV, QIMR Berghofer Institute of Medical Research	(23)
Ganaplacide (formerly KAF156, GNF156)	NCT01753323	II	Uncomplicated P. falciparum and P. vivax malaria adult patients	41	Thailand, Vietnam	Novartis Pharmaceuticals	(24)
GSK3191607	NCT02737007	I	Healthy volunteers	6	United Kingdom	GlaxoSmithKline, Hammersmith Medicines Research	(25)
MMV390048	NCT02230579, NCT02281344, and NCT02554799	I	Healthy volunteers and P. falciparum-infected healthy subjects	59	South Africa, Australia, United Kingdom	MMV, University of Cape Town, Q-Pharm Pty Limited, Richmond Pharmacology Limited	(26)
	NCT02783820 and NCT02783833	I	Healthy volunteers and P. falciparum-infected healthy subjects	33	Australia	MMV, Clinical Network Services (CNS) Pty Ltd., Q-Pharm Pty Limited, QIMR Berghofer Institute of Medical Research	(27)
SAR97276	NCT00739206 and NCT01445938	II	Uncomplicated and severe P. falciparum malaria pediatric and adult patients	113	Benin, Burkina Faso, Gabon, Tanzania, Kenya	Sanofi	(29)
(+)-SJ000557733 (also known as SJ733)	NCT02661373 and NCT02867059	I	Healthy volunteers and P. falciparum-infected healthy subjects	40	United States, Australia	St. Jude Children’s Hospital, MMV, Eisai Inc., Global Health Innovative Technology Fund, QIMR Berghofer Institute of Medical Research, Q-Pharm Pty Limited, Clinical Network Services (CNS) Pty Ltd	(28)
Tafenoquine (formerly SB-252263; WR238605)	NCT02184637	I	Healthy volunteers	24	United States	GlaxoSmithKline, MMV	(30)
Artefenomel plus DSM265	NCT02389348	I and II	P. falciparum-infected healthy subjects	13	Australia	MMV, Q-Pharm Pty Limited, QIMR Berghofer Medical Research Institute, Clinical Network Services (CNS) Pty Ltd	(32)
Artefenomel plus Piperaquine	NCT02083380	II	Uncomplicated P. falciparum malaria pediatric and adult patients	437	Benin, Burkina Faso, Democratic Republic of the Congo;, Gabon, Mozambique, Uganda, Vietnam	MMV	(31)
Ferroquine-artesunate	NCT00563914	I and II	Uncomplicated P. falciparum malaria adult patients	46	Gabon, Kenya	Sanofi	(33)
Ganaplacide-piperaquine	Not provided	I	Healthy volunteers	72	Australia	Novartis Pharmaceuticals	(34)
Methylene blue, artesunate, and amodiaquine	ACTRN12612001298808	I	Healthy volunteers	15	Vietnam	Australian Army Malaria Institute, Vietnam People’s Army	(35)
Tafenoquine-artemether-lumefantrine	NCT02184637	I	Healthy volunteers	22	United States	GlaxoSmithKline, MMV	(30)
Tafenoquine-dihydroartemisinin-piperaquine	NCT02184637	I	Healthy volunteers	24	United States	GlaxoSmithKline, MMV	(30)

a MMV, Medicine for Malaria Venture.

### Monotherapy studies of antimalarial drug candidates.

There were 28 published and completed, unpublished studies that evaluated antimalarial drug candidates as monotherapy for malaria treatment (12–30). Nearly half of these studies recruited Australian populations (*n* = 12). Studies were conducted in healthy volunteers (*n* = 8) (17, 25, 30), induced blood-stage malaria (IBSM) subjects (*n* = 7) (12, 14, 15, 18, 21, 23), a combination of healthy volunteers and IBSM subjects (*n* = 5) (20, 26–28), and malaria patients (*n* = 8) (13, 16, 19, 22, 24, 29). Of the studies involving IBSM subjects and malaria patients, in only four studies were antimalarial drug candidates for treating P. vivax evaluated (14, 16, 22, 24). Among studies in malaria patients, six studies involved adults (13, 16, 19, 22, 24), while the remaining two studies recruited both adults and children (29, 31).

A total of 14 new antimalarial compounds were identified, 9 of which are in phase II (Table 3). The majority of these compounds have activity against asexual blood stages of *Plasmodium* (target candidate profile 1 [TCP-1], 13 compounds). Some of the compounds concomitantly have activity against parasite gametocytes (TCP-5, 9 compounds), hepatic schizonts (TCP-4, 5 compounds), and hypnozoites (TCP-3, 1 compound). Ten compounds exert their antimalarial activity via seven different mechanisms of action, while the mode of action of another four compounds is not well understood. Compounds that inhibit P. falciparum P-type ATPase (*Pf*ATP4), such as cipargamin, GSK3191607, and (+)-SJ000557733, were the most frequently studied (*n* = 7).

**TABLE 3:** Overview of antimalarial compounds in development^*a*^^,^^*e*^

Compound	Phase	Presumed target or mechanism of action	Target candidate profile activities^*b*^	Status in development	Data availability
ACT-451840	I	Unknown	Asexual blood stages, transmission reduction	Active (no progress report in the last 2 yrs)	Detailed summary PK-PD data available from published manuscript (12)
AQ-13	II	Inhibition of heme detoxification	Asexual blood stages	Active	Detailed summary of PK-PD data available from published manuscript (13)
Artefenomel (formerly OZ439)	II	Oxidative stress	Asexual blood stages, transmission reduction	Inactive (formulation challenges)	Detailed summary PK-PD data available from published manuscript (14–16) and the results section on the clinical trial registry (NCT03660839)
Cipargamin (formerly KAE609, NIDT609)	II	*Pf*ATP4 inhibition	Asexual blood stages, transmission reduction	Active	Detailed summary PK-PD data available from published manuscript (17–19) and the result section on the clinical trial registry (NCT03660839)
DSM265	II	*Pf*DHODH inhibition	Asexual blood stages, causal (i.e., pre-erythrocytic) prophylaxis	Inactive (formulation challenges)	Detailed summary PK-PD data available from published manuscript (20–22) and the results section on the clinical trial registry (NCT03637517)
Ferroquine (formerly SSR97193, ferrochloroquine)	II	Inhibition of heme detoxification	Asexual blood stages	Inactive (insufficient level of efficacy as a single-dose cure)	Detailed summary PK-PD data available from published manuscript (23), individual deidentified PK-PD data available from published manuscript (23)
Ganaplacide (formerly KAF156; GNF156)	II	Unknown^*c*^	Asexual blood stages, transmission reduction, causal prophylaxis	Inactive (insufficient level of efficacy as a single-dose cure)	Detailed summary PK-PD data available from published manuscript (24)
GSK3191607	I	*Pf*ATP4 inhibition	Asexual blood stages, transmission reduction	Inactive (short half-life for an oral single-dose cure)	Detailed summary PK-PD data available from published manuscript (25), individual deidentified PK-PD data available through the Clinical Study Data Request repository (https://www.clinicalstudydatarequest.com) (25)
M5717 (formerly DDD107498, DDD498, and MMV121)	I	*Pf*eEF2 inhibition	Asexual blood stages, transmission reduction, causal prophylaxis	Active	Not yet available, manuscript in prepn by sponsor
MMV390048 (also known as MMV048)	II	*Pf*PI4K inhibition	Asexual blood stages, transmission reduction, causal prophylaxis	Inactive (high dose is required as a single-dose cure to achieve efficacy level)	Detailed summary PK-PD data available from published manuscript (26, 27)
MMV688533 (also known as MMV533, SAR441121)	I	Unknown	Asexual blood stages	Active	Not yet available (in recruiting process)
SAR97276	II	Choline uptake inhibition	Asexual blood stages	Inactive (insufficient level of efficacy as a single-dose, once- or twice-daily 3-day regimen)	Detailed summary PK-PD data available from published manuscript (29), individual deidentified PK-PD data available by contacting the lead manuscript authors (29)
(+)-SJ000557733 (also known as SJ733)	I	*Pf*ATP4 inhibition	Asexual blood stages, transmission reduction	Active	Detailed summary PK-PD data available from published manuscript (28), individual deidentified PK-PD data available by contacting the lead manuscript authors (28)
Tafenoquine (formerly SB-252263; WR238605)	II	Unknown	Transmission reduction, causal prophylaxis, relapse prevention	Active	Detailed summary PK-PD data available from published manuscript (30)
ZY-19489 (formerly AZ13721412, MMV674253)	I	Unknown	Asexual blood stages	Active	Not yet available, manuscript in prepn by sponsor
Artefenomel plus DSM265	I	Artefenomel, oxidative stress; DSM265, DHODH inhibition	Asexual blood stages, causal prophylaxis, transmission reduction	Inactive (formulation challenges)	Detailed summary PK-PD data available from published manuscript (32)
Artefenomel-ferroquine	II	Artefenomel, oxidative stress; ferroquine, inhibition of heme detoxification	Asexual blood stages, transmission reduction	Inactive (failed pivotal phase II study)	Detailed summary of PK-PD data available from the result section on the clinical trial registry (NCT03660839), individual deidentified PK-PD data available through the Clinical Study Data Request repository (https://www.clinicalstudydatarequest.com) (NCT03660839)
Artefenomel-piperaquine	II	Artefenomel, oxidative stress; piperaquine, inhibition of heme detoxification	Asexual blood stages, transmission reduction	Inactive (did not reach a satisfactory efficacy level)	Detailed summary PK-PD data available from published manuscript (31)
Ferroquine-artesunate	II	Ferroquine, inhibition of heme detoxification; artesunate, free radical-mediated oxidative stress	Asexual blood stages	Inactive (concerns about the rise of resistance)	Detailed summary PK-PD data available from published manuscript (33)
Ganaplacide-lumefantrine^*d*^	II	Ganaplacide, unknown; lumefantrine, inhibition of β-hematin formation	Asexual blood stages, transmission reduction, causal prophylaxis	Active	Not yet available (in recruiting process)
Ganaplacide-piperaquine	I	Ganaplacide, unknown; piperaquine, inhibition of heme detoxification	Asexual blood stages, transmission reduction, causal prophylaxis	Active	Detailed summary PK-PD data available from published manuscript (34)
Methylene blue with artesunate and amodiaquine	I	Methylene blue, inhibition of heme polymerization mediated by *Pf*GR; artesunate, free radical-media ted oxidative stress; amodiaquine, inhibition of heme detoxification	Asexual blood stages, transmission reduction	Active	Detailed summary PK-PD data available from published manuscript (35)
Ruxolitinib-artemether-lumefantrine	I	Ruxolitinib, JAK inhibitor; artemether, free radical-mediated oxidative stress; lumefantrine, inhibition of β-hematin formation	Asexual blood stages (ruxolitinib as an immune booster)	Active	Not available
Tafenoquine-artemether-lumefantrine	II	Tafenoquine, unknown; artemether, free radical-mediated oxidative stress; lumefantrine, inhibition of β-hematin formation	Asexual blood stages, transmission reduction, causal prophylaxis, relapse prevention	Active	Detailed summary PK-PD data available from published manuscript (30)
Tafenoquine-DHA-piperaquine	II	Tafenoquine, unknown; DHA, free radical-mediated oxidative stress; piperaquine, inhibition of heme detoxification	Asexual blood stages, transmission reduction, causal prophylaxis, relapse prevention	Active	Detailed summary PK-PD data available from published manuscript (30)

a Antimalarial compounds listed are from published and unpublished, completed studies as well as studies that were in the process of recruiting subjects between 1 January 2016 and 28 April 2021.

b Asexual blood stages, TCP-1; relapse prevention, TCP-3; transmission reduction, TCP-5 and TCP-6; causal prophylaxis, TCP-4.

c Decreased susceptibility to ganaplacide is associated with mutations in the *Pfcarl* (cyclic amine resistance locus), *Pfugt* (encodes UDP-galactose transporters), and *Pfact* (encodes acetyl-CoA transporters) genes.

d As solid dispersion formulation.

e *Pf*ATP4, P. falciparum P-type ATPase; *Pf*DHODH, P. falciparum dihydro-orotate dehydrogenase; *Pf*eEF2, P. falciparum translational elongation factor 2; *Pf*PI4K, P. falciparum phosphatidylinositol-4-kinase; *Pf*GR, P. falciparum glutathione reductase; JAK, Janus-associated kinases; DHA, dihydroartemisinin; PK-PD, pharmacokinetics-pharmacodynamics.

### Combination therapy studies of antimalarial drug candidates.

Antimalarial drug candidates as combination therapies were evaluated in nine published and completed, unpublished studies (30–35). Almost half of these studies were conducted in Australia (*n* = 4). Of these studies, four involved healthy volunteers (34, 35), two were undertaken in IBSM subjects (32), and the remaining three recruited malaria patients (31, 33). P. falciparum was the only parasite species investigated in IBSM subjects and malaria patients. Of studies involving malaria patients, two were carried out in both adults and children (31) and one in adults (33).

Ten different combination therapies were identified (Table 3). Of these, combinations of two compounds were evaluated in six studies (31–34). The remaining three studies investigated triple antimalarial combination therapies (30, 35). Most of the studies (*n* = 8) examined the combination of one new antimalarial drug candidate with an on-market compound(s). Only two studies examined a combination of new antimalarial drug candidates. Five nonartemisinin-based combination therapies (non-ACT) were tested in five studies. Artefenomel was frequently investigated as a non-ACT, in combination with DSM265 (32), piperaquine (31), and ferroquine (unpublished). ACTs were examined in four studies, four of which were triple antimalarial combination therapies (30, 35).

### Status of antimalarial drug candidates in the phase I and II drug development pipeline.

The status of antimalarial drug candidates in the phase I and II drug development pipeline is summarized in Table 3. As of 28 April 2021, the antimalarial drug development landscape includes 25 antimalarial drug candidates, of which 14 are active and the remaining 11 are inactive for reasons given in Table 3. Of the 14 confirmed active projects, 8 have been evaluated as monotherapies and another 6 as combination therapies. Although the status of ACT-451840 is active, there has been no progress reported for the last 2 years. The reasons for an inactive status included insufficient efficacy level (*n* = 6), formulation challenge (*n* = 3), short half-life for development of an oral single-dose cure (*n* = 1), and concern about the rise of resistance (*n* = 1).

### Data availability.

Detailed summaries of PK-PD data were available from 24 open-access published manuscripts for 17 antimalarial drug candidates, with 1 study also providing deidentified individual participant data (IPD) (23). In addition to summaries of PK-PD data, deidentified IPD can be requested through the Clinical Study Data Request repository (*n* = 1) (25) or by contacting the corresponding authors (*n* = 2) (28, 29). Of 12 completed, unpublished studies, deidentified IPD sharing was available for three studies through the Clinical Study Data Request repository or stated by the investigators as available upon reasonable request. Results were also posted on clinical trial registries for four completed, unpublished studies.

Most of the published studies investigated oral antimalarial drug candidates, with the exception of two studies in which candidates were delivered by an intravenous or intramuscular route (25, 29) (Table S2 in the supplemental material). Antimalarial drug candidates were tested as single doses (*n* = 20) (12, 14–23, 25–28, 30–32, 34, 35), 3-day regimens (*n* = 2) (13, 33), and both single doses and 3-day regimens (*n* = 2) (24, 29). The majority of antimalarial drug concentrations were measured in plasma (*n* = 22) (12, 14–35). Blood concentrations of antimalarial compounds were determined in two studies (13, 20). All published studies reported PK parameter estimates derived from noncompartmental analysis (12–18, 20–27, 29, 30, 32–35) or *post hoc* empirical Bayesian estimates of population PK models (19, 28, 31). Parasitemia was monitored using microscopy (*n* = 3) (16, 24, 29), qualitative PCR (qPCR; *n* = 10) (12, 14, 15, 18, 20, 21, 23, 27, 28, 32), and both microscopy and qPCR (*n* = 2) (19, 22). Parasitemia clearance curve metrics were mainly estimated following the method described by Marquart et al. (36) (*n* = 10) (12, 14, 15, 18, 20, 21, 23, 27, 28, 32) and Worldwide Antimalarial Resistance Network (WWARN) Parasite Clearance Estimator (37) (*n* = 4) (16, 19, 22, 24). PK-PD models were developed to characterize the relationship between antimalarial drug candidate concentration and parasite clearance in nine studies (12, 14, 15, 18–20, 23, 27, 28).

### Pharmacokinetic and pharmacodynamic properties of new antimalarial compounds.

The PK and PD parameter estimates of new antimalarial compounds are presented in Table S2. The elimination half-life (*t*_½_) ranged from 14.7 (95% confidence interval [CI], 12.1 to 27.1) to 483.9 (95% CI, 352.3 to 664.7) h following a single oral dose administration (12, 14–24, 26–28, 30–32, 34, 35) and 29.9 (95% CI, 19.4 to 40.4) to 92.4 (95% CI, 58.6 to 126.2) h after a 3-day oral regimen (13, 24, 33). PK interactions of three different antimalarial combination therapies were explored in three studies (30, 34, 35). Coadministration of ganaplacide and piperaquine significantly increased maximum concentrations (*C*_max_) of ganaplacide (1.23-fold; 90% CI, 1.10 to 1.37) and piperaquine (1.69-fold; 90% CI, 1.16 to 2.45), with no impact on area under the concentration-time curve (AUC; fold changes were not reported by the authors) (34). The authors concluded that the increase in *C*_max_ for either compound was unlikely to be clinically relevant given the lack of relationship between increased *C*_max_ of each drug and elevated Fridericia’s formula-corrected QT interval (QTcF). While PK of dihydroartemisinin (DHA), piperaquine, artemether, and lumefantrine were not affected by coadministration of tafenoquine, a nonsignificant increase in tafenoquine *C*_max_ (38%; 90% CI, 25 to 52), AUC from 0 h to infinity (AUC_0–∞_) (12%; 90% CI, 1 to 26) and *t*_½_ (29%; 90% CI, 19 to 40) were observed in the presence of DHA-piperaquine (30). The PK profile of tafenoquine was not altered by artemether-lumefantrine coadministration. These PK interactions were not considered clinically relevant, and therefore, no dose adjustment was deemed necessary when coadministering these compounds. Methylene blue significantly increased DHA AUC_0–∞_ (1.05-fold; 90% CI, 1.02 to 1.08) when administered concomitantly with artesunate-amodiaquine but did not influence artesunate, amodiaquine, and desethylamodiaquine PK profiles (35).

The log_10_ parasite reduction rate over 48 h (PRR_48_) for P. falciparum ranged from 1.55 (95% CI, 1.42 to 1.67) to 4.1 (95% CI, 3.7 to 4.4) following a single dose of oral monotherapy (12, 15, 18–23, 27, 28) and 2.71 (95% CI, 2.57 to 2.85) to 4.29 (95% CI, 2.87 to 5.7) following a single dose of oral combination therapy (32). Single-oral-dose monotherapy resulted in parasite clearance half-life (PCt_½_) of 3.4 (95% CI, 1.4 to 7.2) to 9.4 (95% CI, 8.7 to 10.2) h (12, 15, 16, 18, 20–24, 27, 28) and 1.75 (95% CI, 1.57 to 1.97) to 5.33 (95% CI, 5.07 to 5.62) h for a single dose of oral combination therapy (32) against P. falciparum. Upon a single-oral-dose monotherapy, the log_10_ PRR_48_ ranged from 0.9 (95% CI, 0.5 to 1.3) to 1.67 (95% CI, 1.55 to 1.78) (14, 22) and PCt_½_ values were 2.34 (95% CI, 1.24 to 3.88) to 18 (95% CI, 12.1 to 23.9) h (14, 16, 22, 24) in vivax malaria. The clearance rate of P. vivax after a single oral dose of combination therapy was not evaluated in any study. Likewise, there was a paucity of studies investigating parasite clearance rate following a 3-day regimen. A 3-day oral regimen of monotherapy resulted in log_10_ PRR_48_ of 3.18 (range, 1.51 to 3.85) and PCt_½_ of 3.5 (range, 2.8 to 5.1) h against P. falciparum (24) and 3.49 (range, 3.1 to 3.78) and 1.9 (range, 0.9 to 2.7) h against P. vivax (24). There was a negative association between PRR and PCt_½_ (Fig. S1).

This scoping review presents a systematic overview of antimalarial drug candidates that have undergone phase I and II studies in the past 5 years. In this review, we have identified 50 studies, and evidence regarding studies investigating antimalarial drug candidates used as monotherapy and combination therapy, status of antimalarial drug candidates in the drug development pipeline, and data availability were synthesized from 37 published and completed, unpublished studies. It reveals that 14 antimalarial compounds were tested as monotherapy, and 10 different antimalarial combinations were investigated. It highlights that 14 antimalarial candidates are currently active in the drug development pipeline, with detailed summaries of the PK and PD data available for 24 studies.

While almost all published and completed, unpublished studies investigated antimalarial drug candidates for clearance of asexual blood stages (TCP-1), only nine and three studies evaluated compounds with concomitant hepatic schizonticide (TCP-3) and both hepatic schizonticide and hypnozoiticide (TCP-3 and TCP-4) activities, respectively. Although the blood-stage infection is responsible for clinical symptoms, targeting liver-stage parasites presents a promising strategy for malaria eradication, as this stage is a crucial checkpoint in the parasite life cycle. The lack of efficient high-throughput screening assays contributes to the limited development of antimalarial drug candidates against liver schizonts and hypnozoites (38, 39). Beyond liver stages, targeting parasite transmission is another critical step toward malaria eradication. Transmission blocking is achieved either by targeting the mosquito vector (TCP-5) or sexual blood stages (TCP-6). Although concomitant endectocidal activity was not tested in the studies examining compounds, concomitant gametocytocidal or transmission-blocking activity had been characterized in 25 studies testing compounds (40).

Ideally, a combination of at least two antimalarial compounds administered as a single dose should clear asexual blood stages, block transmission, and eliminate hepatic schizonts, including hypnozoites (single-exposure radical cure and prophylaxis [SERCaP]). Achieving cure with a single-dose cure would decrease the cost of treatment and allow directly observed administration, thus ensuring compliance. However, none of the new compounds given as monotherapy were predicted to lead to complete clearance of all asexual and sexual stage parasites with a single dose (10), requiring repeated administration for a complete cure. Therefore, this ambitious target product profile may require multiple exposures of two compounds or a single exposure of three or more antimalarial combinations (10). We found two-thirds of the published and completed, unpublished studies investigated administration of the drug as a monotherapy. Initially characterizing the PK-PD relationship from monotherapy studies is important for guiding dose optimization before being deployed as a combination therapy. Information on contribution of individual drugs and their interactions (i.e., on drug concentration, parasite growth and killing, or both) derived from monotherapy and combination studies is a prerequisite for defining rational dosing regimens of antimalarial combinations (41–44).

Our findings suggest that the current antimalarial drug development pipeline mirrors those of infectious diseases in general. Of 25 projects, 14 were active over the last 5 years. This number is comparable to the success rate of phase I and II for anti-infective medicines (38.4 to 70.1%) (45, 46) and antimalarial compounds in the Medicines for Malaria Venture (MMV) discovery portfolio (60 to 70%) (10). The percentage of antimalarial drug candidates that progress from phase I and II studies to successful product registration ranged from 16% to 30% (10), consistent with the likelihood of approval from phase I and II for infectious diseases (13.2 to 22.8%) (46). Half of the inactive compounds in this review have been associated with poor efficacy where the cure rate at day 28 ranged from 59 to 91% with a single dose (24, 29, 31). These cure rates did not achieve the target efficacy of >95% (10). A similar percentage (48%) was observed for phase II clinical trial failure attributable to efficacy issues between 2013 and 2015 (47).

Given the importance of PK-PD characterization for dose optimization, we have included information on data availability and provided the estimated PK and PD parameters. We identified 25 open-access published manuscripts that provided detailed summaries of PK-PD data. In addition, the findings of four studies have been posted on clinical trial registries. The World Health Organization has outlined the timeline for submission of main findings to be published in a peer-reviewed, open-access journal within 12 to 24 months after completing the trial (https://www.who.int/clinical-trials-registry-platform/reporting-on-findings). Additionally, it is also required to report the key outcomes on the clinical trial registry within 12 months after completion of the trial (https://www.who.int/clinical-trials-registry-platform/reporting-on-findings). Open-access availability of data in the public domain maximizes the benefit of these data to the scientific community. In addition, many government and philanthropic funders require, as a condition of support, that raw data be made available to the scientific community. To the best of our knowledge, there is no repository of PK-PD data of antimalarial drug candidates under investigation in phase I and II studies. We took the initiative to collate this information to help generate insights on how these antimalarial compounds compare against TCP criteria and current therapies.

All of the new antimalarial compounds in these published studies have a long duration of action, with an elimination *t*_½_ ranging from 14.7 to 483.9 h. This is a significant improvement over the short elimination *t*_½_ of artemisinin derivatives (for single doses, artesunate, 0.5 h [48]; artemisinin, 1.8 h [49]; and artemether, 3.1 h [50]; for multiple doses, artesunate, 0.5 h [51]; artemisinin, 1.3 h [52], and artemether, 4.2 h [50]). This is to be expected, as compounds in development have been selected based on their ability to maintain therapeutic concentrations for at least 4 days (10). These values are consistent with the elimination *t*_½_ of amodiaquine (12.4 to 15.6 h), desethylamodiaquine (10 to 12.4 h) (53, 54), lumefantrine (14.2 h) (55), chloroquine (156 h), desethylchloroquine (83 h) (56), mefloquine (200 h) (57), and piperaquine (540 h) (58). In general, the rates of P. falciparum clearance assessed by log_10_ PRR_48_ or PCt_½_ were slightly slower for cipargamin (log_10_ PRR_48_, 3.08 [95% CI, 2.66 to 4.43] to 3.72 [95% CI, 3.44 to 4.2]; PCt_½_, 3.99 [95% CI, 3.79 to 4.21] h), ganaplacide (log_10_ PRR_48_, 3.17 [range, 2.27 to 4.06]; PCt½, 3.4 [range, 1.4 to 7.2] h), artefenomel (log_10_ PRR_48_, 2.2 [95% CI, 2.09 to 2.35] to 4.01 [95% CI, 3.76 to 4.25]; PCt_½_, 3.6 [95% CI, 3.4 to 3.8] to 6.5 [95% CI, 6.2 to 6.9] h), and SJ733 (log_10_ PRR_48_, 2.2 [95% CI, 2.0 to 2.5] to 4.1 [95% CI, 3.7 to 4.4]; PCt_½_, 3.56 [95% CI, 3.29 to 3.88] to 6.47 [95% CI, 5.88 to 7.18] h) and were substantially slower in ACT-451840 (log_10_ PRR_48_, 1.87 [95% CI, 1.75 to 1.98]; PCt_½_, 7.7 [95% CI, 7.3 to 8.3] h), DSM265 (log_10_ PRR_48_, 1.55 [95% CI, 1.42 to 1.67] to 3.9 [95% CI, 2.1 to 5.7]; PCt_½_, 4.9 [95% CI, 3.5 to 6.3] to 9.4 [95% CI, 8.7 to 10.2] h), ferroquine (log_10_ PRR_48_, 2.21 [95% CI, 2.15 to 2.27]; PCt_½_, 6.5 [95% CI, 6.4 to 6.7] h), and MMV048 (log_10_ PRR_48_, 2.3 [95% CI, 2.1 to 2.4] to 2.6 [95% CI, 2.4 to 2.8]; PCt_½_, 5.5 [95% CI, 5.2 to 6.0] to 6.4 [95% CI, 6.0 to 6.9] h) than those of artesunate monotherapy (log_10_ PRR_48_, 4.59 [95% CI, 4.38 to 4.79]; PCt½, 3.2 [95% CI, 3.0 to 3.3] h) (48). These compounds, with the exception of ACT-451840, fulfilled the minimum essential criterion of rapid clearance of parasites at least as fast as mefloquine (log_10_ PRR_48_, 2.2 [95% CI, 2.11 to 2.28] to 2.29 [95% CI, 2.19 to 2.39]) (57).

There are several limitations of our review that warrant care in the interpretation of the findings. Although we made every effort to collate PK-PD data for antimalarial drug candidates undergoing phase I and II investigation, our findings may be impacted by data unavailability due to the delay between study completion and publication. This was mitigated by extracting study results posted on clinical trial registries. Database and clinical trial registry searching was limited to the last 5 years on the basis of the average duration spent by anti-infectives in phase I and II (46). Hence, PK-PD data before this period were not included in this review; such historical data may be valuable in providing additional knowledge of the compounds. Clinical trial registry information such as recruitment status are not updated regularly, which may affect our findings. In one study, it was reported that 31% of clinical trials either had incorrect listed recruitment status or had a delay of recruitment status update of over 1 year (59). We addressed this by checking the date of last update on trial registries and providing a statement if the study status has not been updated for more than 2 years. Study status was not updated for more than 2 years in three studies; these constituted only a small percentage of the included studies (6%). It must be noted that sample sizes of the included studies were small, and the majority of the PK parameter estimates were derived from noncompartmental analysis. Because antimalarial drugs are often reported to have multiple-compartment kinetics, the compound concentration may have declined rapidly to a value below the concentration at half of the maximum effect (EC_50_) before the elimination phase, making it difficult to assess the drug’s potential effect. This was investigated by comparing the compound concentration at the start of the elimination phase and its EC_50_ (reported by the authors or from *in vitro* studies). We found that the compound concentration reached the EC_50_ in 68% of the studies before being eliminated, and we were unable to infer for 29% of the studies due to insufficient information. Another limitation is related to the use of PCt_½_ as a PD measure. In addition to the effect of the drug, host-acquired immunity is an important factor that influences parasite clearance. However, the contribution of immunity on parasite clearance is relatively small, with a maximum shortening of PCt_½_ values <40 min (60). Moreover, 71% of the included studies that reported PCt_½_ were conducted in volunteer-infected studies where acquired immunity plays little or no role. Cytoadherence could also influence the interpretation of the parasite clearance curve following treatment with antimalarial drugs which do not kill ring-form parasites (44). Most of the compounds that reported parasite clearance information in this scoping review have a broad-spectrum activity against blood-stage parasites. Additionally, other measures such as PRR were also used to capture the PD response of antimalarial drugs. PRR cancels out the effect of cytoadherence, as the parasite populations were assessed at the same stages of development separated by one cycle (44).

**Conclusions.** The need for antimalarial compounds with novel modes of action has become a high priority in drug development due to the emergence of multidrug-resistant malaria. The last 5 years have seen a number of antimalarial drug candidates being investigated as monotherapy and in combination with other antimalarial therapies. Some of these compounds have demonstrated promising PK-PD properties, with 14 compounds currently active in the antimalarial drug development landscape. Given that PK-PD data from phase I and II studies are informative for streamlining the progress of antimalarial compounds to the next phase, timely public disclosure of these data is paramount.

## References

[1] World Health Organization. 2020. World malaria report 2020. World Health Organization, Geneva, Switzerland.

[2] World Health Organization. 2015. Global technical strategy for malaria 2016–2030. World Health Organization, Geneva, Switzerland.

[3] Payne D. 1987. Spread of chloroquine resistance in Plasmodium falciparum. Parasitol Today 3:241–246. doi:10.1016/0169-4758(87)90147-5. 15462966

[4] Price RN, von Seidlein L, Valecha N, Nosten F, Baird JK, White NJ. 2014. Global extent of chloroquine-resistant Plasmodium vivax: a systematic review and meta-analysis. Lancet Infect Dis 14:982–991. doi:10.1016/S1473-3099(14)70855-2. 25213732 PMC4178238

[5] Noedl H, Se Y, Schaecher K, Smith BL, Socheat D, Fukuda MM, Artemisinin Resistance in Cambodia 1 (ARC1) Study Consortium. 2008. Evidence of artemisinin-resistant malaria in western Cambodia. N Engl J Med 359:2619–2620. doi:10.1056/NEJMc0805011. 19064625

[6] Dondorp AM, Nosten F, Yi P, Das D, Phyo AP, Tarning J, Lwin KM, Ariey F, Hanpithakpong W, Lee SJ, Ringwald P, Silamut K, Imwong M, Chotivanich K, Lim P, Herdman T, An SS, Yeung S, Singhasivanon P, Day NP, Lindegardh N, Socheat D, White NJ. 2009. Artemisinin resistance in Plasmodium falciparum malaria. N Engl J Med 361:455–467. doi:10.1056/NEJMoa0808859. 19641202 PMC3495232

[7] Ashley EA, Dhorda M, Fairhurst RM, Amaratunga C, Lim P, Suon S, Sreng S, Anderson JM, Mao S, Sam B, Sopha C, Chuor CM, Nguon C, Sovannaroth S, Pukrittayakamee S, Jittamala P, Chotivanich K, Chutasmit K, Suchatsoonthorn C, Runcharoen R, Hien TT, Thuy-Nhien NT, Thanh NV, Phu NH, Htut Y, Han KT, Aye KH, Mokuolu OA, Olaosebikan RR, Folaranmi OO, Mayxay M, Khanthavong M, Hongvanthong B, Newton PN, Onyamboko MA, Fanello CI, Tshefu AK, Mishra N, Valecha N, Phyo AP, Nosten F, Yi P, Tripura R, Borrmann S, Bashraheil M, Peshu J, Faiz MA, Ghose A, Hossain MA, Samad R, Tracking Resistance to Artemisinin Collaboration (TRAC), et al. 2014. Spread of artemisinin resistance in Plasmodium falciparum malaria. N Engl J Med 371:411–423. doi:10.1056/NEJMoa1314981. 25075834 PMC4143591

[8] Phyo AP, Ashley EA, Anderson TJC, Bozdech Z, Carrara VI, Sriprawat K, Nair S, White MM, Dziekan J, Ling C, Proux S, Konghahong K, Jeeyapant A, Woodrow CJ, Imwong M, McGready R, Lwin KM, Day NPJ, White NJ, Nosten F. 2016. Declining efficacy of artemisinin combination therapy against P falciparum malaria on the Thai-Myanmar border (2003–2013): the role of parasite genetic factors. Clin Infect Dis 63:784–791. doi:10.1093/cid/ciw388. 27313266 PMC4996140

[9] van der Pluijm RW, Imwong M, Chau NH, Hoa NT, Thuy-Nhien NT, Thanh NV, Jittamala P, Hanboonkunupakarn B, Chutasmit K, Saelow C, Runjarern R, Kaewmok W, Tripura R, Peto TJ, Yok S, Suon S, Sreng S, Mao S, Oun S, Yen S, Amaratunga C, Lek D, Huy R, Dhorda M, Chotivanich K, Ashley EA, Mukaka M, Waithira N, Cheah PY, Maude RJ, Amato R, Pearson RD, Goncalves S, Jacob CG, Hamilton WL, Fairhurst RM, Tarning J, Winterberg M, Kwiatkowski DP, Pukrittayakamee S, Hien TT, Day NP, Miotto O, White NJ, Dondorp AM. 2019. Determinants of dihydroartemisinin-piperaquine treatment failure in Plasmodium falciparum malaria in Cambodia, Thailand, and Vietnam: a prospective clinical, pharmacological, and genetic study. Lancet Infect Dis 19:952–961. doi:10.1016/S1473-3099(19)30391-3. 31345710 PMC6715822

[10] Burrows JN, Duparc S, Gutteridge WE, Hooft van Huijsduijnen R, Kaszubska W, Macintyre F, Mazzuri S, Mohrle JJ, Wells TNC. 2017. New developments in anti-malarial target candidate and product profiles. Malar J 16:26. doi:10.1186/s12936-016-1675-x. 28086874 PMC5237200

[11] Duffey M, Blasco B, Burrows JN, Wells TNC, Fidock DA, Leroy D. 2021. Assessing risks of Plasmodium falciparum resistance to select next-generation antimalarials. Trends Parasitol 37:709–721. doi:10.1016/j.pt.2021.04.006. 34001441 PMC8282644

[12] Krause A, Dingemanse J, Mathis A, Marquart L, Mohrle JJ, McCarthy JS. 2016. Pharmacokinetic/pharmacodynamic modelling of the antimalarial effect of Actelion-451840 in an induced blood stage malaria study in healthy subjects. Br J Clin Pharmacol 82:412–421. doi:10.1111/bcp.12962. 27062080 PMC4972157

[13] Koita OA, Sangare L, Miller HD, Sissako A, Coulibaly M, Thompson TA, Fongoro S, Diarra Y, Ba M, Maiga A, Diallo B, Mushatt DM, Mather FJ, Shaffer JG, Anwar AH, Krogstad DJ. 2017. AQ-13, an investigational antimalarial, versus artemether plus lumefantrine for the treatment of uncomplicated Plasmodium falciparum malaria: a randomised, phase 2, non-inferiority clinical trial. Lancet Infect Dis 17:1266–1275. doi:10.1016/S1473-3099(17)30365-1. 28916443 PMC5700806

[14] Collins KA, Abd-Rahman AN, Marquart L, Ballard E, Gobeau N, Griffin P, Chalon S, Mohrle JJ, McCarthy JS. 2020. Antimalarial activity of artefenomel against asexual parasites and transmissible gametocytes during experimental blood-stage Plasmodium vivax infection. J Infect Dis doi:10.1093/infdis/jiaa287. PMC8922009 32479608

[15] McCarthy JS, Baker M, O'Rourke P, Marquart L, Griffin P, Hooft van Huijsduijnen R, Mohrle JJ. 2016. Efficacy of OZ439 (artefenomel) against early Plasmodium falciparum blood-stage malaria infection in healthy volunteers. J Antimicrob Chemother 71:2620–2627. doi:10.1093/jac/dkw174. 27272721 PMC4992851

[16] Phyo AP, Jittamala P, Nosten FH, Pukrittayakamee S, Imwong M, White NJ, Duparc S, Macintyre F, Baker M, Mohrle JJ. 2016. Antimalarial activity of artefenomel (OZ439), a novel synthetic antimalarial endoperoxide, in patients with Plasmodium falciparum and Plasmodium vivax malaria: an open-label phase 2 trial. Lancet Infect Dis 16:61–69. doi:10.1016/S1473-3099(15)00320-5. 26448141 PMC4700386

[17] Huskey SE, Zhu CQ, Fredenhagen A, Kuhnol J, Luneau A, Jian Z, Yang Z, Miao Z, Yang F, Jain JP, Sunkara G, Mangold JB, Stein DS. 2016. KAE609 (cipargamin), a new spiroindolone agent for the treatment of malaria: evaluation of the absorption, distribution, metabolism, and excretion of a single oral 300-mg dose of [14C]KAE609 in healthy male subjects. Drug Metab Dispos 44:672–682. doi:10.1124/dmd.115.069187. 26921387

[18] McCarthy JS, Abd-Rahman AN, Collins KA, Marquart L, Griffin P, Kummel A, Fuchs A, Winnips C, Mishra V, Csermak-Renner K, Jain JP, Gandhi P. 2021. Defining the antimalarial activity of cipargamin in healthy volunteers experimentally infected with blood-stage Plasmodium falciparum. Antimicrob Agents Chemother 65:e01423-20. doi:10.1128/AAC.01423-20. 33199389 PMC7849011

[19] Hien TT, White NJ, Thuy-Nhien NT, Hoa NT, Thuan PD, Tarning J, Nosten F, Magnusson B, Jain JP, Hamed K. 2017. Estimation of the in vivo MIC of cipargamin in uncomplicated Plasmodium falciparum malaria. Antimicrob Agents Chemother 61:e01940-16. doi:10.1128/AAC.01940-16. 27872070 PMC5278730

[20] McCarthy JS, Lotharius J, Ruckle T, Chalon S, Phillips MA, Elliott S, Sekuloski S, Griffin P, Ng CL, Fidock DA, Marquart L, Williams NS, Gobeau N, Bebrevska L, Rosario M, Marsh K, Mohrle JJ. 2017. Safety, tolerability, pharmacokinetics, and activity of the novel long-acting antimalarial DSM265: a two-part first-in-human phase 1a/1b randomised study. Lancet Infect Dis 17:626–635. doi:10.1016/S1473-3099(17)30171-8. 28363636 PMC5446412

[21] Collins KA, Ruckle T, Elliott S, Marquart L, Ballard E, Chalon S, Griffin P, Mohrle JJ, McCarthy JS. 2019. DSM265 at 400 milligrams clears asexual stage parasites but not mature gametocytes from the blood of healthy subjects experimentally infected with Plasmodium falciparum. Antimicrob Agents Chemother 63:e01837-18. doi:10.1128/AAC.01837-18. 30858218 PMC6437518

[22] Llanos-Cuentas A, Casapia M, Chuquiyauri R, Hinojosa JC, Kerr N, Rosario M, Toovey S, Arch RH, Phillips MA, Rozenberg FD, Bath J, Ng CL, Cowell AN, Winzeler EA, Fidock DA, Baker M, Mohrle JJ, Hooft van Huijsduijnen R, Gobeau N, Araeipour N, Andenmatten N, Ruckle T, Duparc S. 2018. Antimalarial activity of single-dose DSM265, a novel plasmodium dihydroorotate dehydrogenase inhibitor, in patients with uncomplicated Plasmodium falciparum or Plasmodium vivax malaria infection: a proof-of-concept, open-label, phase 2a study. Lancet Infect Dis 18:874–883. doi:10.1016/S1473-3099(18)30309-8. 29909069 PMC6060173

[23] McCarthy JS, Ruckle T, Djeriou E, Cantalloube C, Ter-Minassian D, Baker M, O'Rourke P, Griffin P, Marquart L, Hooft van Huijsduijnen R, Mohrle JJ. 2016. A phase II pilot trial to evaluate safety and efficacy of ferroquine against early Plasmodium falciparum in an induced blood-stage malaria infection study. Malar J 15:469. doi:10.1186/s12936-016-1511-3. 27624471 PMC5022189

[24] White NJ, Duong TT, Uthaisin C, Nosten F, Phyo AP, Hanboonkunupakarn B, Pukrittayakamee S, Jittamala P, Chuthasmit K, Cheung MS, Feng Y, Li R, Magnusson B, Sultan M, Wieser D, Xun X, Zhao R, Diagana TT, Pertel P, Leong FJ. 2016. Antimalarial activity of KAF156 in falciparum and vivax malaria. N Engl J Med 375:1152–1160. doi:10.1056/NEJMoa1602250. 27653565 PMC5142602

[25] Okour M, Derimanov G, Barnett R, Fernandez E, Ferrer S, Gresham S, Hossain M, Gamo FJ, Koh G, Pereira A, Rolfe K, Wong D, Young G, Rami H, Haselden J. 2018. A human microdose study of the antimalarial drug GSK3191607 in healthy volunteers. Br J Clin Pharmacol 84:482–489. doi:10.1111/bcp.13476. 29168205 PMC5809343

[26] Sinxadi P, Donini C, Johnstone H, Langdon G, Wiesner L, Allen E, Duparc S, Chalon S, McCarthy JS, Lorch U, Chibale K, Mohrle J, Barnes KI. 2020. Safety, tolerability, pharmacokinetics, and antimalarial activity of the novel Plasmodium phosphatidylinositol 4-kinase inhibitor MMV390048 in healthy volunteers. Antimicrob Agents Chemother 64:e01896-19. doi:10.1128/AAC.01896-19. 31932368 PMC7179259

[27] McCarthy JS, Donini C, Chalon S, Woodford J, Marquart L, Collins KA, Rozenberg FD, Fidock DA, Cherkaoui-Rbati MH, Gobeau N, Mohrle JJ. 2020. A phase 1, placebo-controlled, randomized, single ascending dose study and a volunteer infection study to characterize the safety, pharmacokinetics, and antimalarial activity of the Plasmodium phosphatidylinositol 4-kinase inhibitor MMV390048. Clin Infect Dis 71:e657–e664. doi:10.1093/cid/ciaa368. 32239164 PMC7744986

[28] Gaur AH, McCarthy JS, Panetta JC, Dallas RH, Woodford J, Tang L, Smith AM, Stewart TB, Branum KC, Freeman BB, III, Patel ND, John E, Chalon S, Ost S, Heine RN, Richardson JL, Christensen R, Flynn PM, Van Gessel Y, Mitasev B, Mohrle JJ, Gusovsky F, Bebrevska L, Guy RK. 2020. Safety, tolerability, pharmacokinetics, and antimalarial efficacy of a novel Plasmodium falciparum ATP4 inhibitor SJ733: a first-in-human and induced blood-stage malaria phase 1a/b trial. Lancet Infect Dis 20:964–975. doi:10.1016/S1473-3099(19)30611-5. 32275867

[29] Held J, Supan C, Salazar CLO, Tinto H, Bonkian LN, Nahum A, Sie A, Abdulla S, Cantalloube C, Djeriou E, Bouyou-Akotet M, Ogutu B, Mordmuller B, Kreidenweiss A, Siribie M, Sirima SB, Kremsner PG. 2017. Safety and efficacy of the choline analogue SAR97276 for malaria treatment: results of two phase 2, open-label, multicenter trials in African patients. Malar J 16:188. doi:10.1186/s12936-017-1832-x. 28472957 PMC5418711

[30] Green JA, Mohamed K, Goyal N, Bouhired S, Hussaini A, Jones SW, Koh GC, Kostov I, Taylor M, Wolstenholm A, Duparc S. 2016. Pharmacokinetic interactions between tafenoquine and dihydroartemisinin-piperaquine or artemether-lumefantrine in healthy adult subjects. Antimicrob Agents Chemother 60:7321–7332. doi:10.1128/AAC.01588-16. 27697758 PMC5119013

[31] Macintyre F, Adoke Y, Tiono AB, Duong TT, Mombo-Ngoma G, Bouyou-Akotet M, Tinto H, Bassat Q, Issifou S, Adamy M, Demarest H, Duparc S, Leroy D, Laurijssens BE, Biguenet S, Kibuuka A, Tshefu AK, Smith M, Foster C, Leipoldt I, Kremsner PG, Phuc BQ, Ouedraogo A, Ramharter M, Group O-PS, OZ-Piperaquine Study Group. 2017. A randomised, double-blind clinical phase II trial of the efficacy, safety, tolerability and pharmacokinetics of a single dose combination treatment with artefenomel and piperaquine in adults and children with uncomplicated Plasmodium falciparum malaria. BMC Med 15:181. doi:10.1186/s12916-017-0940-3. 28988541 PMC5632828

[32] McCarthy JS, Ruckle T, Elliott SL, Ballard E, Collins KA, Marquart L, Griffin P, Chalon S, Mohrle JJ. 2019. A single-dose combination study with the experimental antimalarials artefenomel and DSM265 to determine safety and antimalarial activity against blood-stage Plasmodium falciparum in healthy volunteers. Antimicrob Agents Chemother 64:e01371-19. doi:10.1128/AAC.01371-19. 31685476 PMC7187626

[33] Supan C, Mombo-Ngoma G, Kombila M, Ospina Salazar CL, Held J, Lell B, Cantalloube C, Djeriou E, Ogutu B, Waitumbi J, Otsula N, Apollo D, Polhemus ME, Kremsner PG, Walsh DS. 2017. Phase 2a, open-label, 4-escalating-dose, randomized multicenter study evaluating the safety and activity of ferroquine (SSR97193) plus artesunate, versus amodiaquine plus artesunate, in African adult men with uncomplicated Plasmodium falciparum malaria. Am J Trop Med Hyg 97:514–525. doi:10.4269/ajtmh.16-0731. 28722611 PMC5544076

[34] Leong FJ, Jain JP, Feng Y, Goswami B, Stein DS. 2018. A phase 1 evaluation of the pharmacokinetic/pharmacodynamic interaction of the anti-malarial agents KAF156 and piperaquine. Malar J 17:7. doi:10.1186/s12936-017-2162-8. 29304859 PMC5756412

[35] Anh CX, Chavchich M, Birrell GW, Van Breda K, Travers T, Rowcliffe K, Lord AR, Shanks GD, Edstein MD. 2020. Pharmacokinetics and ex vivo antimalarial activity of artesunate-amodiaquine plus methylene blue in healthy volunteers. Antimicrob Agents Chemother 64:e01441-19. doi:10.1128/AAC.01441-19. 31907186 PMC7038242

[36] Marquart L, Baker M, O'Rourke P, McCarthy JS. 2015. Evaluating the pharmacodynamic effect of antimalarial drugs in clinical trials by quantitative PCR. Antimicrob Agents Chemother 59:4249–4259. doi:10.1128/AAC.04942-14. 25963983 PMC4468738

[37] Flegg JA, Guerin PJ, White NJ, Stepniewska K. 2011. Standardizing the measurement of parasite clearance in falciparum malaria: the parasite clearance estimator. Malar J 10:339. doi:10.1186/1475-2875-10-339. 22074219 PMC3305913

[38] Campo B, Vandal O, Wesche DL, Burrows JN. 2015. Killing the hypnozoite - drug discovery approaches to prevent relapse in Plasmodium vivax. Pathog Glob Health 109:107–122. doi:10.1179/2047773215Y.0000000013. 25891812 PMC4455353

[39] Leroy D, Campo B, Ding XC, Burrows JN, Cherbuin S. 2014. Defining the biology component of the drug discovery strategy for malaria eradication. Trends Parasitol 30:478–490. doi:10.1016/j.pt.2014.07.004. 25131411

[40] Yahiya S, Rueda-Zubiaurre A, Delves MJ, Fuchter MJ, Baum J. 2019. The antimalarial screening landscape - looking beyond the asexual blood stage. Curr Opin Chem Biol 50:1–9. doi:10.1016/j.cbpa.2019.01.029. 30875617 PMC6591700

[41] Dini S, Zaloumis S, Cao P, Price RN, Fowkes FJI, van der Pluijm RW, McCaw JM, Simpson JA. 2018. Investigating the efficacy of triple artemisinin-based combination therapies for treating Plasmodium falciparum malaria patients using mathematical modeling. Antimicrob Agents Chemother 62:e01068-18. doi:10.1128/AAC.01068-18. 30150462 PMC6201091

[42] Dini S, Zaloumis SG, Price DJ, Gobeau N, Kummel A, Cherkaoui M, Moehrle JJ, McCarthy JS, Simpson JA. 2021. Seeking an optimal dosing regimen for OZ439/DSM265 combination therapy for treating uncomplicated falciparum malaria. J Antimicrob Chemother 76:2325–2334. doi:10.1093/jac/dkab181. 34179977 PMC8361368

[43] Hastings IM, Hodel EM. 2014. Pharmacological considerations in the design of anti-malarial drug combination therapies - is matching half-lives enough? Malar J 13:62. doi:10.1186/1475-2875-13-62. 24552440 PMC3975950

[44] White NJ. 2013. Pharmacokinetic and pharmacodynamic considerations in antimalarial dose optimization. Antimicrob Agents Chemother 57:5792–5807. doi:10.1128/AAC.00287-13. 24002099 PMC3837842

[45] Wong CH, Siah KW, Lo AW. 2019. Estimation of clinical trial success rates and related parameters. Biostatistics 20:273–286. doi:10.1093/biostatistics/kxx069. 29394327 PMC6409418

[46] Thomas D, Chancellor D, Micklus A, LaFever S, Hay M, Chaudhuri S, Bowden R, Lo AW. 2021. Clinical development success rates and contributing factors 2011–2020. Biotechnology Innovation Organization, Informa Pharma Intelligence, Quantitative Life Sciences, Washington, DC.

[47] Harrison RK. 2016. Phase II and phase III failures: 2013–2015. Nat Rev Drug Discov 15:817–818. doi:10.1038/nrd.2016.184. 27811931

[48] Watts RE, Odedra A, Marquart L, Webb L, Abd-Rahman AN, Cascales L, Chalon S, Rebelo M, Pava Z, Collins KA, Pasay C, Chen N, Peatey CL, Mohrle JJ, McCarthy JS. 2020. Safety and parasite clearance of artemisinin-resistant Plasmodium falciparum infection: a pilot and a randomised volunteer infection study in Australia. PLoS Med 17:e1003203. doi:10.1371/journal.pmed.1003203. 32822347 PMC7444516

[49] De Vries PJ, Tran KD, Nguyen XK, Le Nguyen B, Pham TY, Dao DD, Van Boxtel CJ, Kager PA. 1997. The pharmacokinetics of a single dose of artemisinin in patients with uncomplicated falciparum malaria. Am J Trop Med Hyg 56:503–507. doi:10.4269/ajtmh.1997.56.503. 9180598

[50] Na Bangchang K, Karbwang J, Thomas CG, Thanavibul A, Sukontason K, Ward SA, Edwards G. 1994. Pharmacokinetics of artemether after oral administration to healthy Thai males and patients with acute, uncomplicated falciparum malaria. Br J Clin Pharmacol 37:249–253. doi:10.1111/j.1365-2125.1994.tb04271.x. 8198933 PMC1364755

[51] Saunders D, Khemawoot P, Vanachayangkul P, Siripokasupkul R, Bethell D, Tyner S, Se Y, Rutvisuttinunt W, Sriwichai S, Chanthap L, Lin J, Timmermans A, Socheat D, Ringwald P, Noedl H, Smith B, Fukuda M, Teja-Isavadharm P. 2012. Pharmacokinetics and pharmacodynamics of oral artesunate monotherapy in patients with uncomplicated Plasmodium falciparum malaria in western Cambodia. Antimicrob Agents Chemother 56:5484–5493. doi:10.1128/AAC.00044-12. 22869581 PMC3486599

[52] Gordi T, Huong DX, Hai TN, Nieu NT, Ashton M. 2002. Artemisinin pharmacokinetics and efficacy in uncomplicated-malaria patients treated with two different dosage regimens. Antimicrob Agents Chemother 46:1026–1031. doi:10.1128/AAC.46.4.1026-1031.2002. 11897585 PMC127081

[53] Rijken MJ, McGready R, Jullien V, Tarning J, Lindegardh N, Phyo AP, Win AK, Hsi P, Cammas M, Singhasivanon P, White NJ, Nosten F. 2011. Pharmacokinetics of amodiaquine and desethylamodiaquine in pregnant and postpartum women with Plasmodium vivax malaria. Antimicrob Agents Chemother 55:4338–4342. doi:10.1128/AAC.00154-11. 21709098 PMC3165320

[54] Tarning J, Chotsiri P, Jullien V, Rijken MJ, Bergstrand M, Cammas M, McGready R, Singhasivanon P, Day NP, White NJ, Nosten F, Lindegardh N. 2012. Population pharmacokinetic and pharmacodynamic modeling of amodiaquine and desethylamodiaquine in women with Plasmodium vivax malaria during and after pregnancy. Antimicrob Agents Chemother 56:5764–5773. doi:10.1128/AAC.01242-12. 22926572 PMC3486620

[55] Jain JP, Leong FJ, Chen L, Kalluri S, Koradia V, Stein DS, Wolf MC, Sunkara G, Kota J. 2017. Bioavailability of lumefantrine is significantly enhanced with a novel formulation approach, an outcome from a randomized, open-label pharmacokinetic study in healthy volunteers. Antimicrob Agents Chemother 61:e00868-17. doi:10.1128/AAC.00868-17. 28630183 PMC5571342

[56] Abd-Rahman AN, Marquart L, Gobeau N, Kummel A, Simpson JA, Chalon S, Mohrle JJ, McCarthy JS. 2020. Population pharmacokinetics and pharmacodynamics of chloroquine in a Plasmodium vivax volunteer infection study. Clin Pharmacol Ther 108:1055–1066. doi:10.1002/cpt.1893. 32415986 PMC7276750

[57] McCarthy JS, Marquart L, Sekuloski S, Trenholme K, Elliott S, Griffin P, Rockett R, O'Rourke P, Sloots T, Angulo-Barturen I, Ferrer S, Jimenez-Diaz MB, Martinez MS, Hooft van Huijsduijnen R, Duparc S, Leroy D, Wells TN, Baker M, Mohrle JJ. 2016. Linking murine and human Plasmodium falciparum challenge models in a translational path for antimalarial drug development. Antimicrob Agents Chemother 60:3669–3675. doi:10.1128/AAC.02883-15. 27044554 PMC4879391

[58] Hoglund RM, Workman L, Edstein MD, Thanh NX, Quang NN, Zongo I, Ouedraogo JB, Borrmann S, Mwai L, Nsanzabana C, Price RN, Dahal P, Sambol NC, Parikh S, Nosten F, Ashley EA, Phyo AP, Lwin KM, McGready R, Day NP, Guerin PJ, White NJ, Barnes KI, Tarning J. 2017. Population pharmacokinetic properties of piperaquine in falciparum malaria: an individual participant data meta-analysis. PLoS Med 14:e1002212. doi:10.1371/journal.pmed.1002212. 28072872 PMC5224788

[59] Jones CW, Safferman MR, Adams AC, Platts-Mills TF. 2017. Discrepancies between ClinicalTrials.gov recruitment status and actual trial status: a cross-sectional analysis. BMJ Open 7:e017719. doi:10.1136/bmjopen-2017-017719. PMC5652524 29025842

[60] Ataide R, Ashley EA, Powell R, Chan JA, Malloy MJ, O'Flaherty K, Takashima E, Langer C, Tsuboi T, Dondorp AM, Day NP, Dhorda M, Fairhurst RM, Lim P, Amaratunga C, Pukrittayakamee S, Hien TT, Htut Y, Mayxay M, Faiz MA, Beeson JG, Nosten F, Simpson JA, White NJ, Fowkes FJ. 2017. Host immunity to Plasmodium falciparum and the assessment of emerging artemisinin resistance in a multinational cohort. Proc Natl Acad Sci USA 114:3515–3520. doi:10.1073/pnas.1615875114. 28289193 PMC5380044

[61] Peters MDJ, Marnie C, Tricco AC, Pollock D, Munn Z, Alexander L, McInerney P, Godfrey CM, Khalil H. 2020. Updated methodological guidance for the conduct of scoping reviews. JBI Evid Synth 18:2119–2126. doi:10.11124/JBIES-20-00167. 33038124

[62] Tricco AC, Lillie E, Zarin W, O'Brien KK, Colquhoun H, Levac D, Moher D, Peters MDJ, Horsley T, Weeks L, Hempel S, Akl EA, Chang C, McGowan J, Stewart L, Hartling L, Aldcroft A, Wilson MG, Garritty C, Lewin S, Godfrey CM, Macdonald MT, Langlois EV, Soares-Weiser K, Moriarty J, Clifford T, Tuncalp O, Straus SE. 2018. PRISMA Extension for Scoping Reviews (PRISMA-ScR): checklist and explanation. Ann Intern Med 169:467–473. doi:10.7326/M18-0850. 30178033

